# Effect of Health Insurance Uptake on Hesitancy toward COVID-19 Vaccines in Nigeria: A Recursive Bivariate Probit and Decomposition Estimation

**DOI:** 10.3390/ijerph20032566

**Published:** 2023-01-31

**Authors:** Abayomi Samuel Oyekale

**Affiliations:** Department of Agricultural Economic and Extension, North-West University, Mafikeng Campus, Mmabatho 2735, South Africa; abayomi.oyekale@nwu.ac.za or asoyekale@gmail.com

**Keywords:** health insurance, moral hazard, COVID-19, vaccination, recursive probit model

## Abstract

Moral hazard remains one of the major challenges of health insurance administration. This paper recursively analyzed the effect of health insurance on the willingness to take COVID-19 vaccines in Nigeria. The data comprised 1892 unvaccinated respondents in the 2021/2022 National Longitudinal Phone Survey (NLPS). The data were analyzed with Coban’s recursive probit regression and decomposition approaches. The results revealed that 5.87% were health insured, and 7.93% were willing to take COVID-19 vaccines. Health insurance uptake significantly increased (*p* < 0.05) with an adult being the decision-maker on vaccination, requiring family planning, and urban residence, while it reduced with loss of jobs and residence in the southeast and southwest zones. In addition, health insurance significantly (*p* < 0.01) increased the willingness to take COVID-19 vaccines, along with each adult, all adults, and households’ heads being the major vaccination decision-makers, loss of jobs, and support for making COVID-19 vaccines compulsory. The average treatment effects (ATEs) and average treatment effect on the treated (ATET) of health insurance were significant (*p* < 0.01), with positive impacts on willingness to be vaccinated. It was concluded that policy reforms to promote access to health insurance would enhance COVID-19 vaccination in Nigeria. In addition, hesitancy toward COVID-19 vaccines can be reduced by targeting adults and household heads with adequate information, while health insurance uptake should target southern states and rural areas.

## 1. Introduction

Coronaviruses are epidemiologically associated with upper respiratory infections of significant severity and are a public health concern [1,2,3,4]. They have been linked to diagnosed pneumonia, asthmatic complications, and chronic bronchitis in children, adults, and elderly people [5,6,7,8,9]. After the first characterization of the human coronaviruses in the 1960s, the fifth human coronavirus, known as SARS CoV, was characterized in 2002 [10]. The sixth virus, known as Middle East Respiratory Syndrome (MERS), was characterized in 2013, while SARS-CoV-2 is the seventh virus, which was discovered in 2019, and is also responsible for the COVID-19 pandemic [10,11]. SARS-CoV-2 is among the deadliest coronaviruses ever discovered among humans [12]. As of 31 October 2022, they have been globally linked to more than 6.5 million human deaths among more than 627 million diagnosed cases [13].

Vaccination has been amplified as one of the major ways of reversing SARS-CoV-2 infection waves, the severity of illness, and their associated mortality. This can be understood from its being one of the foremost pillars of preventive medicine, which has, over the decades, offered significant efficiency in healthcare service delivery [14]. Some estimates have shown that administered vaccines prevent more than 3 million deaths annually that would have resulted from vaccine-preventable diseases, the majority of which affect neonatal and children under the age of five [15,16]. It should be emphasized that besides improved sanitation and clean water, vaccination has been described as the most cost-effective public health intervention in the world [14,17,18]. However, the acceptability of vaccines remains a major healthcare service delivery problem in many developing countries. Across the world, misinformation on COVID-19 vaccines is affecting their acceptability [19]. Although 12,830,378,906 doses of COVID-19 vaccines have been globally administered as of 26 October 2022 [13], many developing countries are yet to vaccinate a significant number of their eligible citizens [20,21].

African countries generally had a very slow roll-out of COVID-19 vaccines [22]. Besides the supply problem, the demand for COVID-19 vaccines was also marred by hesitancy that was promoted by misinformation. Across many African countries, low perceptions of vaccine efficacy and safety are the major drivers of hesitancy [23]. In Nigeria, the roll-out of COVID-19 vaccines was very slow. Available statistics revealed that as of 19 March 2022, one year after its commencement, only 20,157,050 people were fully vaccinated, while 10,925,624 were partially vaccinated [24]. More importantly, Nigeria was unable to meet the 70% vaccination target that was set by the World Health Organization (WHO) for its member countries. At the expiration of the June 2022 deadline, partially vaccinated persons stood at 11,830,098, while 25,812,414 were fully vaccinated [25].

The low coverage of COVID-19 vaccination among Nigerians raises some fundamental concerns. More specifically, some demographic characteristics have been documented as correlates of vaccine hesitancy [26,27,28,29,30,31,32]. These include education [26,31], age [27,32], adverse side effects [28,29], gender [30], marital status [32], employment status [26,31], religion [26,31,32], ethnicity [30], needing medical services [27], conspiracy theory [28], and knowing someone who was infected with COVID-19 [31]. Besides these variables, there is also the need to empirically understand the effect of health insurance uptake on the willingness to take COVID-19 vaccines. Therefore, there may be an increase in the utilization of preventive healthcare services such as vaccination due to the uptake of health insurance [33]. On the contrary, the existence of moral hazards among health-insured individuals may promote some level of reluctance toward vaccination if the costs of treatment against sicknesses are fully covered by the insurance policy [34,35].

Some studies found some associations between access to health insurance and utilization of preventive healthcare services [36,37,38,39]. In a study by Jerant et al. [40], uptake of health insurance was significantly associated with increased utilization of preventive healthcare services, such as influenza vaccination and screening for colorectal, cervical, breast, and prostate cancers. However, they did not find a significant association between access to health insurance and engagement in healthy lifestyles. Some authors have also found significant associations between health insurance uptake and the acceptability of COVID-19 vaccines [41,42,43]. In another study, the acceptability of COVID-19 vaccines was not significantly influenced by the uptake of health insurance [44]. The type of health insurance was reported by Lu et al. [45] as a significant factor influencing the completion of childhood immunization. Other authors have also reported the statistical significance of some demographic variables in explaining the acceptability of COVID-19 vaccines. These include education [46,47], age [46,48], gender [49,50], ethnicity [49], sector of residence [51], marital status [52], being previously diagnosed with COVID-19 [53], and perception of vaccines’ side effects and their effectiveness [54,55].

This paper seeks to fill a major gap in the literature by recursively analyzing the impact of health insurance uptake on the willingness to be vaccinated against COVID-19 in Nigeria. The study is among the few that have taken this important perspective. The study also presents some uniqueness from the proposed analytical approaches through the adoption of the bivariate probit decomposition framework that was recently developed by Coban [56,57].

## 2. Materials and Methods

### 2.1. The Data and Sampling Procedures

Secondary data were utilized for this study. The data were sourced from the second phase of the National Longitudinal Phone Survey (NLPS). The data were collected by the National Bureau of Statistics (NBS) in conjunction with the World Bank, which also provided the funding. The major objective of the second phase was to have some evidence-based monitoring of the welfare impacts of some idiosyncratic and covariate shocks among Nigerian households. The survey, being a continuation of the first phase, also adopted the sampling frame of the 2018/2019 GHS-Panel surveys without discriminating against those who participated in the first phase. There were 4976 nationally representative households in the sampling frame, while 4440 provided their phone numbers. Out of the households that could be contacted telephonically, 2797 households were reached to participate in the surveys, of which 2750 successfully participated [58]. In order to ensure representativeness, sampling weights were generated for each of the respondents, and these were used for data analyses.

The first round of the surveys was conducted between 29 November 2021 and 16 January 2022. In addition, the second round of the surveys took place between 29 January 2022 and 14 February 2022. The respondents were interviewed by some trained enumerators using computer-assisted telephone interviews (CATIs). The enumerators were mandated to either place phone calls from their homes to selected respondents using the phone tablets that were provided by the NBS or make the calls from the NBS calling centers. Upon completion, the data were captured and uploaded to the NBS server. These were vetted by some assigned supervisors for quality assurance through identification of outliers and wrong entries [44]. Verbal consent to participate in the surveys was obtained from the respondents, who must be adult (18 years and above) members of selected households. In addition, upon successful completion of the interviews, each participant was given N=1000.00 airtime to compensate for their involvement in the surveys [58].

### 2.2. Analytical Models

This study adopted the recently developed STATA module to estimate recursive probit model using the “rbiprobit” command [56,57]. This model was estimated for a segment of the respondents who had not taken any dose of COVID-19 vaccines. The proposed model assumed that health insurance, being a treatment variable, is likely to be endogenous. Based on the assumption of significant correlation between the error terms in the insurance and vaccination models, the model was recursively estimated. Following Coban [56], two structural equations can be presented:

(1) $$y_{1}^{*}=x^{\prime }\beta +\alpha y_{2}+e_{1},\;\;y_{1}=1[y_{1}^{*}>0]$$

(2) $$y_{2}^{*}=x^{\prime }\omega +e_{2},\;\;y_{2}=1[y_{2}^{*}>0]$$

In Equations (1) and (2), $y_{1}^{*}$ is the willingness to be vaccinated against COVID-19 (yes = 1, 0 otherwise), $y_{2}^{*}$ is treatment and endogenous regressor, which is uptake of health insurance (yes = 1, 0 otherwise), and $x^{\prime }$ is the vector of other included explanatory variables (see Table 1). β, α, and ω are the vectors of the estimated parameters. Moreover, $e_{1}$ and $e_{2}$ are the error terms for Equations (1) and (2), respectively. It should also be noted that $\left(\begin{array}{c} e_{1}\\ e_{2} \end{array}\right)\sim N\left[\left(\begin{array}{c} 0\\ 0 \end{array}\right),\left(\begin{array}{cc} 1 & \rho \\ \rho & 1 \end{array}\right)\right]$, with ρ being the correlation coefficient between the error terms. The software generated Wald test statistics to conclude whether any significant correlation exists between the two error terms. If the null hypothesis of no correlation is accepted, the model can be estimated using the conventional probit model without any consideration of endogeneity. Some variables could not be used as independent variables due to missing data values.

According to Greene [59], the average treatment effect (ATE) of health insurance can be expressed as:

(3) $$\mathrm{ATE}=\frac{1}{w}\overset{n}{\underset{i=1}{\sum }}w_{i}[\Phi \left(x_{i}^{\prime }\beta +\alpha \right)-\Phi \left(x_{i}^{\prime }\beta \right)]$$

In Equation (3), w  is the total sampling weight, $w_{i}$ is the sampling weight of ith respondent, and *n* is the number of observations.

The average treatment effect on the treated (ATET) is expressed as:

(4) $$\mathrm{ATET}=\frac{1}{w_{2}}\overset{n_{2}}{\underset{i=1}{\sum }}w_{i}[\Phi \left(\frac{x_{i}^{\prime }\beta +\alpha -\rho x_{i}^{\prime }\omega }{\sqrt{1-\rho^{2}}}\right)-\Phi \left(\frac{x_{i}^{\prime }\beta -\rho x_{i}^{\prime }\omega }{\sqrt{1-\rho^{2}}}\right)]\;\forall y_{2i}=1$$

The average treatment effect on conditional probability (ATEC)

(5) $$\mathrm{ATEC}=\frac{1}{w_{2}}\sum_{i=1}^{n_{2}}w_{i}[\frac{\Phi_{2}\left(x_{i}^{\prime }\beta +\alpha \rho x_{i}^{\prime }\omega \right)}{\Phi (x_{i}^{\prime }\omega )}-\frac{\Phi_{2}\left(x_{i}^{\prime }\beta -x_{i}^{\prime }\omega -\rho \right)}{\Phi (-x_{i}^{\prime }\omega )}]$$

In Equations (4) and (5), $w_{2}$ is the total sampling weight for the treated, and $n_{2}$ is the number of treated observations.

In addition, Greene [59] decomposed the total marginal effects into direct and indirect components. Each of the independent variables in Equations (1) and (2) can be decomposed for these impacts. By designating $x_{1T}$ for Equation (1) (treatment equation) and $x_{1E}$ for Equation (2) (endogenous equation), the decomposition of the marginal parameter into its direct and indirect components can be implemented. If the variable is in continuous form, this can be expressed as [59]:

(6) $$ME=\frac{\delta Pr}{\delta \left(\begin{array}{c} x_{1T}\\ x_{1E} \end{array}\right)}=\overset{\frac{\delta Pr}{\delta x_{1T}}}{\underset{direct\;effect}{︸}}+\overset{\frac{\delta Pr}{\delta x_{1E}}}{\underset{indirect\;effect}{︸}}$$

Discrete independent variables in both the treatment and endogenous equations can be decomposed by following Hasebe [60] and Edwards et al. [61]:

(7) $$ME=\overset{{\left|Pr\right|}_{x_{1T=1}-}{\left|Pr\right|}_{x_{1T=0}}}{\underset{diriect\;effect}{︸}}\;+\;\overset{{\left|Pr\right|}_{x_{1E=1}-}{\left|Pr\right|}_{x_{1E=0}}}{\underset{indirect\;effect}{︸}}$$

## 3. Results

### 3.1. Demographic Characteristics of the Respondents

Table 2 shows the respondents’ demographic variables across their vaccine hesitancy and health insurance status. It reveals that 92.07% of respondents were not willing to be vaccinated, and 94.13% were not health insured. Moreover, out of the vaccine-hesitant respondents, 5.34% were health insured compared with 12.00% for the vaccine-unhesitant. Moreover, out of the health-uninsured respondents, 7.41% were willing to be vaccinated compared with 16.22% for the health-insured. Urban residents accounted for 38.79% of the total respondents. In addition, 62.28% of vaccine-hesitant respondents were from urban areas, while 59.46% of health-insured respondents were from urban areas. The samples were dominated by males at 80.92%, while 81.06% of the vaccine-hesitant were males. Health-insured respondents were relatively younger, with a mean age of 47.88 years compared with 49.75 years for the uninsured. However, there was a very small difference between the average ages of the vaccine-hesitant (49.66 years) and vaccine-unhesitant (49.34 years).

The table further shows that 31.24% of all respondents had lost one job or the other since the inception of the pandemic. Furthermore, 34.00% of respondents who were willing to be vaccinated had lost their jobs. Among the health-insured respondents, 26.13% had lost their jobs. Based on the nature of medical services that were needed during COVID-19, 29.97% of the combined respondents needed non-COVID-19 medical services for adult members. This can be compared with 35.14% of health-insured respondents who needed medical services for adult members. In addition, 19.50% of all respondents required non-COVID-19 medical services for children, which can also be compared with 28.83% for those who were health insured. Table 2 further reveals that household heads were the major decision-makers on COVID-19 vaccination issues, with 51.53% in the combined data. Among health-insured respondents, 58.56% indicated that heads of households were the decision-makers on vaccination. In addition, compulsory COVID-19 vaccination was advocated by 67.02% of the combined respondents, which can be compared with 80.67% of those who were willing to be vaccinated.

### 3.2. Health Insurance and Correlates of COVID-19 Vaccination Status

The results of the recursive probit regression analysis are presented in Table 3. The table contains the estimated parameter for health insurance uptake, which is the treatment and endogenous regressor. It also contains the parameters for willingness to be vaccinated. The estimated models produced good fits for the data based on the computed Wald chi-square statistic that was significant (*p* < 0.01). Similarly, the test statistic for rho equaled zero and was significant (*p* < 0.01). This implies that the error terms of the recursively estimated health insurance and willingness to be vaccinated models are correlated. The computed rho was −0.8885, depicting a strong negative correlation.

#### 3.2.1. Determinants of Health Insurance Subscription

Table 3 shows that for model 1, two of the zonal variables were significant (*p* < 0.05). The parameters of southeast (−0.3998) and southwest (−0.6243) were negative, implying that holding other variables constant, respondents from the southwest and southeast zones had a lower likelihood of being health insured when compared with those from northcentral. The parameters of the employment-related variables in the model—worked for pay (0.2511) and lost jobs (−0.2455)—were significant (*p* < 0.01). These results imply that the respondents who were working for pay and those who had lost their jobs during COVID-19 had higher and lower likelihoods of being health insured, respectively. In addition, the urban residence parameter (0.5793) was significant (*p* < 0.01). This shows that in comparison with those from rural areas, the respondents who were residing in urban areas had a higher likelihood of being health insured. Table 3 further shows that the parameter of family planning (0.8806) was significant (*p* < 0.05) and shows that those who needed family planning services had a higher probability of being health insured. Based on households’ COVID-19 vaccination decision-makers, the parameter of each adult as a decision-maker was 0.3307 and significant (*p* < 0.05). This shows that respondents from households where decisions to be vaccinated were taken by each adult had a significantly higher likelihood of being health insured.

#### 3.2.2. Health Insurance Effect on Willingness to Be Vaccinated and Other Correlates

Table 3 also shows the estimated parameters for the willingness to be vaccinated (model 2). The results indicated that health insurance had a positive parameter (0.1265) that was significant (*p* < 0.01). This shows that the respondents who were health-insured had a higher probability of willingness to take COVID-19 vaccines. The parameter of the lost job variable was also positive (0.2227) and significant (*p* < 0.01). This implies that those respondents who had lost their jobs during the COVID-19 pandemic had a higher probability of willingness to be vaccinated (*p* < 0.01). The parameter of vaccination compulsion was positive (1.0808) and significant (*p* < 0.01). This shows that those respondents who were in support of making COVID-19 vaccination compulsory had a higher probability of willingness to be vaccinated. The parameters of COVID-19 vaccination decision-makers were positive and significant (*p* < 0.01), with each adult, all adults, and household heads having 1.3866, 1.6535, and 1.6338, respectively. These results imply that those respondents from households where each adult was the main decision-maker on COVID-19 vaccination had a higher probability of willingness to be vaccinated. In addition, the respondents who indicated that all adults jointly made COVID-19 vaccination decisions had a higher probability of willingness to be vaccinated. In addition, the respondents from households where the heads were the main COVID-19 vaccination decision-makers had a higher probability of willingness to be vaccinated.

#### 3.2.3. Direct and Indirect Parameter Decomposition and Treatment Effects

Table 4 shows the results of marginal parameter decomposition into their direct, indirect, and total effect components. It shows that except for the southeast and northeast zones, the direct effect marginal parameters of all the zonal variables were negative. Residence in the southeast zone significantly (*p* < 0.05) and indirectly reduced the probability of willingness to be vaccinated by 2.63%. Moreover, residence in the southsouth and southwest zones significantly (*p* < 0.05) and directly reduced the probabilities of willingness to take COVID-19 vaccines by 3.41% and 2.81%, respectively. Under the total effect, only the marginal parameter of the southwest zone showed significance (*p* < 0.05). This implied that residence in the southwest zone decreased the probability of willingness to be vaccinated by 4.31%.

Some of the decomposed marginal parameters of “worked for pay” and “lost job” variables were significant (*p* < 0.05). Specifically, the respondents who worked for pay had their probability of willingness to be vaccinated significantly increased by 2.10% (*p* < 0.05). Moreover, loss of job directly increased the probability of willingness to be vaccinated by 0.40%, while it indirectly reduced it by 1.77%. The marginal parameters of urban residence for the indirect and total marginal contributions were statistically significant (*p* < 0.01) and positive. These results showed that urban residence indirectly increased the probability of willingness to take COVID-19 vaccines by 4.18%. However, when considered in totality, residence in urban areas increased the probability of willingness to take COVID-19 vaccines by 4.00%.

The direct marginal parameter of “make vaccines mandatory” was significant (*p* < 0.01). This result showed that agreeing on making COVID-19 vaccines compulsory directly increased the probability of willingness to take COVID-19 vaccines by 1.96%. Furthermore, the results indicated that the decomposed indirect marginal parameters for needing family planning services were significant (*p* < 0.05). These results implied that needing family planning services increased the willingness to be vaccinated indirectly by 6.35%. The total marginal parameter of non-COVID-19 pharmacy services was significant (*p* < 0.05). This implied that needing non-COVID-19 pharmacy services increased the probability of willingness to take COVID-19 vaccines by 3.00%.

Moreover, the estimated marginal parameters for vaccination decision-makers were significant for each adult and household head. Each adult being the decision-maker directly and indirectly increased the probability of willingness to take COVID-19 vaccines by 3.18% and 3.77%, respectively. Therefore, each adult being the decision-maker increased the probability of willingness to take COVID-19 vaccines by 6.96%. The marginal parameters for household heads being the decision-makers were positive and statistically significant (*p* < 0.05). These results implied that household heads being vaccination decision-makers directly and indirectly increased the probability of willingness to take COVID-19 vaccines by 5.18% and 4.08%, respectively. However, household heads being the vaccination decision-makers increased the probability of willingness to take COVID-19 vaccines by 9.26%.

Table 5 shows the results of the treatment effects of health insurance on the probability of willingness to take COVID-19 vaccines. The estimated parameters showed statistical significance across all three treatment impacts (*p* < 0.05). The ATE of 0.3649 implied that the respondents had their probability of willingness to take COVID-19 vaccines increased by an average of 36.49% due to being health insured. The ATET parameter of 0.5789 implied that health-insured households had their probability of being vaccinated increased by 57.89% more than it would have been if they were not health insured. The ATEC parameter of −0.0654 implied that respondents had their probability of being health insured decreased by an average of 6.54% due to their willingness to take COVID-19 vaccines.

## 4. Discussion

Most of the respondents were not health insured. This agrees with the fact that health insurance uptake in Nigeria is still very low. Therefore, most of the people still rely on out-of-pocket payments for medical fees. Low coverage of health insurance may have resulted from a lack of trust in the workability of the scheme, which initially targeted government workers [62,63]. Some statistics have revealed that only about 3% of Nigerians are covered by health insurance because the majority of those outside formal government employment are unable to pay the required monthly premiums [64,65]. Moreover, although the federal government decentralized the implementation of NHIS to the state government in 2014, subscription to health insurance has been partly low due to low confidence in the workability of the schemes [66]. Therefore, because low health insurance coverage impedes Nigeria’s aspiration to achieve universal health coverage (UHC) [67], a new health insurance bill has been signed into law [64,68]. If successfully implemented, the new bill, which, in principle, makes health insurance mandatory for all Nigerians, will provide a viable platform to achieve the UHC [69].

The result further confirmed the role of health insurance in promoting vaccination against COVID-19. It has been emphasized that health insurance can promote pro-health behavior through the utilization of healthcare services [33], while insured individuals may also engage in morally hazardous behavior by avoiding vaccination [34]. The finding is generally in agreement with the expectation that health insurance can facilitate the utilization of preventive medical services [36,37,38,39,40]. Similarly, the result agrees with the findings of some authors who found health insurance to increase the likelihood of taking COVID-19 vaccines [41,42,43], although it is contrary to the finding of Ku [44].

As expected, urban residents had a higher probability of being health insured. However, urban residence did not influence the willingness to be vaccinated. Moreover, respondents who were working had a higher probability of being health insured and willing to be vaccinated. However, those who had lost their jobs had a lower probability of being insured but a higher probability of willingness to be vaccinated. These findings can be explained by the fact that the implementation of the health insurance scheme started among government workers who were largely in urban areas. These people were also less likely to have lost their jobs because of the pandemic. The findings agree with those of some previous studies [70,71,72,73,74,75] but are contrary to that of Aregbesola et al. [76].

In conformity with some previous studies [70,71], regional and zonal differences exist in access to health insurance in Nigeria. Therefore, the uptake of health insurance was significantly influenced by some zonal variables. Specifically, southern respondents had a lower probability of being health insured. These findings agree with those of Aregbesola et al. [76] and emphasize some zonal differences in access to health insurance, which may have been influenced by awareness and workability perception factors. It should also be emphasized that access to health insurance by northerners may have been boosted by a pilot project on a health insurance scheme for vulnerable families that was implemented in Sokoto state and funded by the United Nations Sustainable Development Programme (SDG) Joint Fund [77]. More importantly, the responses of state governments to the federal government’s promoted health insurance initiatives differ. A good example is the fact that only 19 states have commenced implementation of the directive from the federal government to establish their own State Health Insurance Schemes [62,78].

Moreover, the results showed a very low willingness to take COVID-19 vaccines across every geopolitical zone. However, the southwest zone had the highest percentage of respondents willing to take the vaccines. These results confirm the fact that hesitance toward COVID-19 vaccines remains a major bottleneck to vaccine uptake in Nigeria [79]. The results are also in direct opposition to some previous findings in which willingness to be vaccinated against COVID-19 was initially high [27]. Therefore, the growing hesitance toward COVID-19 vaccines can be attributed to several factors such as misinformation [19,79,80,81] and safety and effectiveness concerns [23,81,82].

The need for medical services due to the presence of some immunocompromised health conditions can influence the decision to be health insured or willing to take COVID-19 vaccines [83,84]. Among the healthcare services that were required by households during the pandemic, family planning showed significance. This finding indicates that those who required family planning services had a higher probability of being health insured. Although the linkage between health insurance and the utilization of family planning services is not well studied in the literature, there have been some positive findings on the increased likelihood of health-insured women utilizing more family planning services [85,86,87,88]. Increased utilization of family planning can have a significant impact on several components of maternal and child health [89], with a drastic reduction in unwanted pregnancies, child mortality, maternal fertility, and maternal mortality. It should also be emphasized that many Nigerians seek prescriptions from pharmacies on issues pertaining to family planning and other health-related issues. This emphasizes the relevance of prescription medicines in healthcare administration and total medical expenditures [90,91]. In Nigeria, it is well acknowledged that health-insured patients pay a very low amount, which is just 10 percent of the total cost [92].

Individuals who agreed with making COVID-19 vaccines mandatory had a higher probability of willingness to be vaccinated. Due to persistent hesitancy, the Nigerian government proposed the compulsory vaccination of workers in the public sector. Agreeing with compulsory vaccination is expected to produce a positive attitude toward COVID-19 vaccination [93]. Although the constitutionality of mandatory COVID-19 vaccination has been extensively argued [94,95], the provision of a conducive environment to all citizens through a timely response to the pandemic is also among the mandates of every government. Therefore, the basic interpretations of the constitutionality of mandatory COVID-19 vaccination are ultimately embedded in which side this is viewed. Finally, the households whose decisions on COVID-19 vaccination were taken by household heads, all adults, and each adult had a higher probability of willingness to be vaccinated. In addition, those with each adult deciding on vaccination had a higher probability of being health insured. It should be noted that in many cases, the decision to be health insured by household members is taken by the household heads. Moreover, based on adjudged vaccine safety, household heads or adult members may be the ultimate decision-makers on COVID-19 vaccination.

## 5. Conclusions

The policy significance of analyzing the impact of health insurance on COVID-19 vaccination decisions cannot be overemphasized. This is a vital issue in Nigeria’s administration of healthcare services due to concurrent hesitancy toward vaccination and very low coverage of health insurance. Beyond addressing the COVID-19 pandemic through vaccination, this study also possesses some direct linkages to the global goals of the UHC to which Nigeria subscribes. This study has made a significant contribution to the existing literature by being one of the few to recursively analyze the effect of health insurance on willingness to be vaccinated against COVID-19 through a recently proposed decomposition and impact assessment approach. The results have established a positive effect of health insurance on willingness to be vaccinated. This has confirmed pro-health behavior among the health insured contrary to our expectation of moral hazards. In addition, the finding reemphasizes the need for government to intensify efforts at ensuring more coverage of health insurance among Nigerian rural dwellers. More importantly, barriers to health insurance and COVID-19 vaccination should be evaluated on a zonal basis, given some existing cultural and socioeconomic diversities in Nigeria. More importantly, by targeting the adult population and household heads, the government should utilize existing media settings in every geopolitical zone to address barriers to health insurance and every pending misinformation and disinformation on COVID-19 vaccines. In addition, health insurance treatment coverage should be expanded to ensure the integration of all family planning and the purchase of all kinds of medication.

## Figures and Tables

**Table 1:** Coding formats of the independent variables.

Variable	Coding Format
Geopolitical Zones	
Northeast	yes = 1, 0 otherwise
Northwest	yes = 1, 0 otherwise
Southeast	yes = 1, 0 otherwise
Southsouth	yes = 1, 0 otherwise
Southwest	yes = 1, 0 otherwise
Urban residence	yes = 1, 0 otherwise
Gender	male = 1, 0 otherwise
Age	years
Work for pay	yes = 1, 0 otherwise
Lost jobs during pandemic	yes = 1, 0 otherwise
Needed medical services during COVID-19	
Needed COVID-19-related services	yes = 1, 0 otherwise
Needed family planning services	yes = 1, 0 otherwise
Needed non-COVID-19 vaccination services	yes = 1, 0 otherwise
Needed non-COVID-19 maternal services	yes = 1, 0 otherwise
Needed non-COVID-19 childcare services	yes = 1, 0 otherwise
Needed non-COVID-19 adult care services	yes = 1, 0 otherwise
Needed non-COVID-19 emergency services	yes = 1, 0 otherwise
Needed pharmacy and chemist services	yes = 1, 0 otherwise
Decision-maker on COVID-19 vaccination	
Each member	yes = 1, 0 otherwise
All adults	yes = 1, 0 otherwise
Household heads	yes = 1, 0 otherwise
Vaccination should be mandatory	yes = 1, 0 otherwise

**Table 2:** Percentages and means of respondents’ demographic variables across vaccine hesitancy and health insurance status.

Variable	Vaccine Unwilling	Vaccine Willing	Health Uninsured	Health Insured	All
Number of respondents	1742	150	1781	111	1892
*Percentages*					
Willing to be vaccinated	0.00	100.00	7.41	16.22	7.93
Health insured	5.34	12.00	0.00	100.00	5.87
*Geopolitical zone*					
Northcentral	14.98	22.67	15.44	18.02	15.59
Northeast	16.93	12.00	16.45	18.02	16.54
Northwest	15.50	9.33	14.21	27.93	15.01
Southeast	21.01	13.33	20.94	11.71	20.4
Southsouth	15.73	13.33	15.67	13.51	15.54
Southwest	15.84	29.33	17.29	10.81	16.91
Other demographic variables					
Urban	37.72	51.33	37.51	59.46	38.79
Rural	62.28	48.67	62.49	40.54	61.21
Male	81.06	79.33	80.57	86.49	80.92
Female	18.94	20.67	19.43	13.51	19.08
Worked for pay	78.82	85.33	78.83	87.39	79.33
Lost job	31.00	34.00	31.56	26.13	31.24
Required healthcare services					
COVID-19-related	0.92	0.00	0.67	3.6	0.85
Family planning	0.57	0.67	0.34	4.5	0.58
Non-COVID-19 vaccination	3.73	2.67	3.31	9.01	3.65
Non-COVID-19 maternal	3.39	4.00	2.98	10.81	3.44
Non-COVID-19 child	19.63	18.00	18.92	28.83	19.5
Non-COVID-19 adult	30.25	26.67	29.65	35.14	29.97
Non-COVID-19 emergency	1.03	2.00	1.01	2.7	1.11
Non-COVID-19 pharmacy	3.44	3.33	3.03	9.91	3.44
Vaccination decision-makers					
Someone else decides	32.26	2.67	30.43	21.62	29.92
Each adult decides	12.11	30.00	13.48	14.41	13.53
All adults decide	4.42	12.00	5.00	5.41	5.02
Household heads decide	51.21	55.33	51.09	58.56	51.53
Make vaccines mandatory	65.84	80.67	67.32	62.16	67.02
*Mean*					
Age of respondents	49.66	49.34	49.75	47.88	49.64

**Table 3:** Parameters of the recursively estimated probit model.

Variables	Model 1. Prediction of Health Insurance		Model 2. Prediction of Willingness to Be Vaccinated	
	Parameter	Standard Error	Parameter	Standard Error
Health insurance			1.6925 ***	0.1738
*Geopolitical zones*				
Northeast zone	0.0183	0.1672	0.1265	0.1203
Northwest zone	0.2864	0.1548	−0.0218	0.1201
Southeast zone	−0.3998 **	0.1741	−0.1037	0.1142
Southsouth zone	−0.1283	0.1645	−0.2006	0.1184
Southwest zone	−0.6243 ***	0.1777	0.0899	0.1185
*Demographic variables*				
Gender	0.0443	0.1337	0.0605	0.0883
Age	−0.0032	0.0030	0.0022	0.0020
Worked for pay	0.2511 ***	0.1276	0.1581	0.0844
Lost job	−0.2455 ***	0.1054	0.2227 ***	0.0730
Urban residents	0.5793 ***	0.1030	−0.1004	0.0755
Make vaccines mandatory	−0.1800	0.1044	1.0808 ***	0.0759
*Needed medical services*				
COVID-19-related	0.4395	0.3816	−0.1802	0.3702
Family planning	0.8806 **	0.4388	−0.8748	0.4886
Non-COVID-19 vaccination	−0.0059	0.2524	0.1448	0.2026
Non-COVID-19 maternal health	0.2712	0.2259	−0.3687	0.1913
Non-COVID-19 child care	0.1295	0.1152	−0.0313	0.0871
Non-COVID-19 adult care	0.1149	0.1038	0.0894	0.0742
Non-COVID-19 emergency	0.1218	0.3473	−0.0535	0.3135
Non-COVID-19 pharmacy	0.3672	0.2193	0.1946	0.2037
*Vaccination decision-maker*				
Each adult	0.3307 **	0.1658	1.3866 ***	0.1135
All adults together	0.1552	0.2371	1.6535 ***	0.1604
Household heads	0.1618	0.1208	1.6338 ***	0.0871
Constant	−1.8796 ***	0.2622	−2.1778 ***	0.1890
Atanrho			−1.4148 ***	0.3101
Rho			−0.8885	0.0653
*Number of observations*	1892			
Wald chi2(45)	906.24 ***			
Wald test of rho = 0	20.819 ***			

Note: *** significant at 1% level; ** significant at 5% level.

**Table 4:** Decomposition of the marginal parameters into their direct and indirect components.

Variables	Direct Effect	Indirect Effect	Total Effect
*Geopolitical zones*			
Northeast zone	0.0023	−0.0004	0.0019
Northwest zone	−0.0038	0.0016	−0.0022
Southeast zone	0.0328	−0.0263 **	0.0065
Southsouth zone	−0.0341 ***	0.0048	−0.0293
Southwest zone	−0.0281 **	−0.0150	−0.0431 ***
Demographic variables			
Gender	0.0011	0.0032	0.0043
Age	0.0000	−0.0002	−0.0002
Worked for pay	0.0029	0.0181	0.0210 **
Lost job	0.0040 ***	−0.0177 **	−0.0137
Urban residents	−0.0018	0.0418 ***	0.0400 ***
Make vaccines mandatory	0.0196 ***	−0.0130	0.0066
*Needed medical services*			
COVID-19-related	−0.0033	0.0317	0.0284
Family planning	−0.0159	0.0635 **	0.0476
Non-COVID-19 vaccination	0.0026	−0.0004	0.0022
Non-COVID-19 maternal health	−0.0067	0.0195	0.0128
Non-COVID-19 child care	−0.0006	0.0093	0.0087
Non-COVID-19 adult care	0.0016	0.0083	0.0099
Non-COVID-19 emergency	−0.0010	0.0088	0.0078
Non-COVID-19 pharmacy	0.0035	0.0265	0.0300 **
*Vaccination decision-maker*			
Each adult	0.0318 ***	0.0377 ***	0.0695 ***
All adults together	0.0238	0.0095	0.0333
Household heads	0.0518 ***	0.0408 **	0.0926 ***

Note: *** significant at 1% level; ** significant at 5% level.

**Table 5:** Treatment effects of health insurance on vaccination decisions.

Treatment Effects	Willing to Be Vaccinated	
	Parameter	Standard Error
Average treatment effect (ATE)	0.3649 ***	0.0279
Average treatment effect on the treated (ATET)	0.5789 ***	0.0581
Average treatment effect on conditional probability (ATEC)	−0.0654 **	0.0302

Note: *** significant at 1% level; ** significant at 5% level.

## Data Availability

The data used for this study are in the public domain, available online: https://microdata.worldbank.org/index.php/catalog/4444 (accessed on 20 September 2022).
